# A Systematic Comparison of Differential Metabolites and Functional Pathways in the Gut Contents of Yaks and Plateau Yellow Cattle Across Altitudinal Gradients

**DOI:** 10.3390/vetsci13101063

**Published:** 2026-10-09

**Authors:** Dan Li, Jian Zhang, Liping Zhang, Xinsheng Liu, Ping Chen

**Affiliations:** 1 State Key Laboratory for Animal Disease Control and Prevention, Lanzhou Veterinary Research Institute, College of Veterinary Medicine, Lanzhou University, Chinese Academy of Agricultural Sciences, Lanzhou 730046, China; 2 Gansu Province Research Center for Basic Disciplines of Pathogen Biology, Lanzhou 730046, China; 3 Institute of Livestock, Grass, and Green Agriculture, Gansu Academy of Agricultural Sciences, Lanzhou 730070, China

**Keywords:** yak, plateau yellow cattle, gut contents, untargeted metabolomics

## Abstract

The yak (*Bos grunniens*) is a crucial species adapted to the extreme high-altitude environment of the Tibetan Plateau. However, a systematic understanding of how gut metabolic homeostasis contributes to this adaptation, particularly across altitudinal gradients and in comparison with sympatric species, remains limited. This study employed untargeted metabolomics to profile the intestinal contents of high-altitude yaks (HYs, 3800 m), low-altitude yaks (LYs, 2500 m), and plateau yellow cattle (PC, 3500 m) from the Gannan region. Multivariate analysis revealed distinct clustering of the three groups, with PLS-DA model validation parameters (R_2_Y(cum) > 0.997, Q_2_(cum) > 0.990), confirming significant species-specific and altitude-adaptive differentiation in the gut metabolome. A suite of differentially abundant metabolites (DAMs) was identified: HYs were enriched with stress- and inflammation-related molecules (e.g., prostaglandin E2, uric acid-indicative of oxidative stress and inflammatory responses) and steroid metabolites; LYs showed a profile oriented towards basic anabolism and antioxidant defense (e.g., L-galactono-1,4-lactone, allantoin); PC were characterized by unique lipid derivatives (e.g., 5-hexyl-2-furanhexanoic acid) and fungal-origin metabolites. Pathway enrichment analysis pinpointed Linoleic acid metabolism as one of the most significantly enriched pathways, with key hydroxyoctadecadienoic acids (HODEs) and related oxylipins significantly upregulated in HYs. Other significantly enriched pathways included Tryptophan metabolism, ABC transporters, and Protein digestion and absorption. Our findings suggest that high-altitude adaptation in yaks is associated with remodeling of the intestinal metabolic network. The identified signature metabolites and consistently enriched candidate pathways provide novel insights into potential host–microbiome-associated mechanisms and establish a metabolic foundation for the conservation of plateau livestock genetic resources and the development of precision husbandry strategies. However, these findings should be interpreted as hypothesis-generating because altitude, species, diet, and environment were not fully decoupled.

## Simple Summary

Yaks are well adapted to the cold, low-oxygen environment of the Tibetan Plateau, but how their gut metabolism supports this adaptation is not fully understood. In this study, we compared the small molecules present in the gut contents of yaks living at high altitude, yaks living at lower altitude, and plateau yellow cattle. We found that the three groups had clearly different metabolic profiles. High-altitude yaks were enriched in molecules related to stress and inflammation, low-altitude yaks showed a profile oriented toward basic metabolism and antioxidant defense, and plateau yellow cattle had unique lipid-related and fungal-derived metabolites. Linoleic acid metabolism was the most consistently enriched pathway, with oxidized lipid products elevated in high-altitude yaks. These findings suggest that high-altitude adaptation in yaks involves remodeling of the gut metabolic network and provide candidate metabolic clues for the conservation of plateau livestock genetic resources and precision husbandry.

## 1. Introduction

The Gannan Tibetan Autonomous Prefecture is located on the northeastern edge of the Qinghai–Tibet Plateau, characterized by year-round high-altitude, cold, and hypoxic conditions. Despite this, yaks (*Bos grunniens*) play an irreplaceable role in local herders’ livelihoods and ecological stability owing to their exceptional high-altitude adaptability [1]. The plateau yellow cattle, a unique breed shaped by long-term natural selection and artificial breeding, is also well adapted to the harsh alpine climate. Together, these two species represent important genetic resources for the Gannan region. Therefore, a thorough understanding of their high-altitude adaptation mechanisms is a key research direction for the development of plateau animal husbandry.

The gut is a vital organ for nutrient regulation in yaks and plays a decisive role in their health and breeding. A first-ever single-cell atlas of the yak gastrointestinal tract has been constructed, identifying the transcription factors HNF4A and SREBF2 as core regulators of the energy metabolism program in gastrointestinal epithelial cells. These two factors work synergistically to regulate cellular glycogen synthesis and reserve as well as lipid transmembrane transport, thereby maintaining energy homeostasis under hypoxic conditions [2]. Nutrition and energy metabolism are central to the regulation of rumen and gut function in yaks. Glutamine has been shown to activate p300-mediated histone lysine lactylation, which upregulates ion transporter expression and repairs rumen epithelial damage caused by feed restriction [3]. The damage mechanisms differ when energy substrates are lacking: glucose deficiency primarily induces oxidative stress, apoptosis, and tight junction disruption via the p38 MAPK/JNK pathway [4], whereas glutamine deficiency is more associated with inflammatory responses. Regarding major diseases, subacute ruminal acidosis (SARA) results from the synergistic effects of short-chain fatty acids (affecting purine metabolism and the cytoskeleton), low pH (disrupting complement function and RNA degradation), and LPS (activating the TNF/MAPK pathway), collectively leading to mitochondrial dysfunction [5]. Furthermore, growth retardation is directly linked to impaired ATP synthesis in rumen epithelial cells, involving downregulation of key enzymes in ketone body synthesis and propionate metabolism, along with compromised mitochondrial function [6]. The above studies represent mechanistic investigations of the yak digestive tract, highlighting an urgent need for large-scale sequencing studies to broadly identify important targets regulating yak intestinal physiology.

There is a mutual interaction between the rumen/gut microbiome and the host in yaks. In terms of seasonal adaptation, cold-season grazing yaks exhibit altered rumen microbial communities (e.g., an increased Firmicutes/Bacteroidetes ratio), enrichment of VFA-producing and arginine synthesis genes, and concurrent upregulation of host epithelial genes involved in VFA absorption and transport, forming an energy compensation network of “microbial fermentation—host absorption” [7]. In summer, microbial diversity is higher, with functions shifting toward amino acid and vitamin metabolism [8]. Regarding developmental patterns, rumen microbial colonisation occurs in stages and shows significant synergy with dynamic changes in host mineral element metabolism genes [9]. Under pathological conditions, diarrhoeal calves display gut microbiota dysbiosis, characterised by reduced diversity, increased pathogenic bacteria, and enrichment of pro-inflammatory functional genes [10]. Moreover, nutritional intervention studies have shown that lactic acid bacteria supplementation optimises the rumen microbiota of yaks fed high-concentrate diets (increasing beneficial bacteria and reducing endotoxin-producing bacteria) and alleviates energy metabolism disorders by modulating host metabolites and activating the AMPK pathway [11]. While these studies have employed omics technologies to investigate environmental adaptation, physiological regulation, disease mechanisms, and nutritional interventions, and have revealed some genetic regulatory patterns of adaptation, there remains a lack of metabolomic studies specifically on yak gut contents.

Although previous studies have revealed aspects of yak high-altitude adaptation at genomic, transcriptomic, and microbiome levels, the gut-content metabolome remains poorly characterized, especially in comparisons that simultaneously consider altitudinal gradients and sympatric plateau cattle. The importance of this study is fourfold. First, it compares high-altitude yaks, low-altitude yaks, and plateau yellow cattle within one framework, allowing altitude gradients and species genetic background to be considered together. Second, it focuses on the gut-content metabolome, which remains less characterized than genomic, transcriptomic, rumen microbiota, or tissue metabolomic data in yaks. Third, it identifies linoleic acid metabolism as a consistently enriched candidate pathway, pyrimidine metabolism as a species-specific pathway in plateau yellow cattle versus low-altitude yaks, and suggests possible roles for tryptophan metabolism and ABC transporters. Fourth, these findings provide candidate metabolic signatures for plateau livestock genetic resource conservation, precision husbandry, and high-altitude hypoxia-related metabolic research. However, this design should be regarded as a hypothesis-generating comparative framework rather than a design that fully decouples altitude, species, diet, and environmental effects.

## 2. Materials and Methods

### 2.1. Sample Collection

In October 2024, adult Gannan native yaks and plateau yellow cattle were selected from Xiahe County, Gannan Tibetan Autonomous Prefecture, Gansu Province, China. Samples were collected in Xiahe County, Gannan Tibetan Autonomous Prefecture. All animals were clinically healthy, in good body condition, with no history of disease, and aged 4–5 years. All animals were female and non-pregnant/non-lactating. Three groups were established, each containing six biological replicates: high-altitude yak group (HY; sampling altitude 3800 m; body weight 280 ± 25 kg), low-altitude yak group (LY; sampling altitude 2500 m; body weight 275 ± 22 kg), and plateau yellow cattle group (PC; sampling altitude 3500 m; body weight 290 ± 28 kg). HYs and PC were raised under the same local traditional grazing system with free access to natural alpine pasture. The feeding time was in 2023, with the same pasture type, dominant vegetation, supplementary feeding, and water source. If LYs differed from HYs in diet or environmental exposure, this difference was recorded and treated as a potential confounder. Animals were euthanized by intravenous injection of sodium pentobarbital (120 mg/kg) (Sigma-Aldrich, St. Louis, MO, USA). The colon intestinal contents were collected within 10 min after slaughter, immediately labelled, snap-frozen in liquid nitrogen, and stored at −80 °C. All samples were processed using identical procedures. All experimental procedures strictly followed the Chinese Standards for the Use and Care of Experimental Animals and were approved by the Animal Ethics and Welfare Committee of the Institute of Livestock, Grass, and Green Agriculture, Gansu Academy of Agricultural Sciences (ethical approval number: GAAS-2024003, approved on 19 April 2024). Study timeline: ethical approval was obtained on 19 April 2024; field sampling was completed in October 2024; LC–MS/GC–MS data acquisition, peak extraction, QC filtering, missing-value imputation, MSI-based annotation, database matching, and multivariate statistics were performed from late 2024 to 2025; pathway enrichment, figure preparation, and manuscript drafting followed. The interval between sampling and publication reflects the time required for untargeted metabolomics annotation and standard peer review, not a delay in study conduct. Detailed animal and sampling metadata, including species, altitude, age, sex, physiological status, body weight, and sampling location, are provided in Supplementary Table S1.

### 2.2. Sample Pretreatment and Metabolite Extraction

Sample consistency was recorded as [solid/semi-solid]. After thawing on ice, all samples were homogenized using the same protocol to minimize handling variation. All samples were processed using identical procedures, including time from slaughter to freezing, homogenization, snap-freezing, and storage at −80 °C. Frozen intestinal contents were thawed on ice. An accurately weighed 100 mg (±0.5 mg) of each sample was placed into a pre-cooled 2 mL grinding tube. Two zirconium oxide grinding beads were added, followed by 1 mL of pre-cooled extraction solvent consisting of methanol (Methanex Corporation, Vancouver, BC, Canada):water (4:1, *v*/*v*) containing 0.1 μg/mL L-2-chlorophenylalanine (Sigma-Aldrich, St. Louis, MO, USA) as an internal standard to monitor extraction efficiency. The mixture was homogenised using a high-throughput tissue grinder (Scientz-48, Ningbo Scientz Biotechnology Co., Ltd., Ningbo, China) at 60 Hz for 3 min at low temperature. The homogenate was then extracted in an ultrasonic water bath at 4 °C for 30 min, followed by centrifugation at 13,000 rpm for 15 min at 4 °C. An 800 μL aliquot of the supernatant was carefully transferred to a new autosampler vial. A portion (e.g., 200 μL) was directly used for liquid chromatography-mass spectrometry (LC-MS) analysis. The remaining supernatant was vacuum freeze-dried. The dried residue was derivatised with 80 μL of methoxyamine hydrochloride reagent (Tokyo Chemical Industry Co., Ltd., Tokyo, Japan) at 37 °C for 90 min, followed by derivatisation with 80 μL of BSTFA (containing 1% TMCS) (Sigma-Aldrich, St. Louis, MO, USA) at 70 °C for 60 min for gas chromatography-mass spectrometry (GC-MS) analysis.

### 2.3. Instrumental Analysis and Data Parameters

Chromatographic conditions: An Agilent 1290 Infinity II UHPLC system (Agilent Technologies, Santa Clara, CA, USA) was used. Chromatographic separation was performed on a Waters ACQUITY UPLC HSS T3 column (Waters Corporation, Milford, MA, USA) (2.1 mm × 100 mm, 1.8 μm) maintained at 40 °C. Mobile phase A was 0.1% formic acid in water, and mobile phase B was 0.1% formic acid (Sigma-Aldrich, St. Louis, MO, USA) in acetonitrile (Merck KGaA, Darmstadt, Germany). The gradient elution program was as follows: 0–2 min, 2% B; 2–15 min, B increased linearly from 2% to 98%; 15–17 min, held at 98% B; 17–17.1 min, B decreased rapidly from 98% to 2%; 17.1–20 min, re-equilibrated to initial conditions. The flow rate was 0.3 mL/min, and the injection volume was 2 μL.

Mass spectrometric conditions: An Agilent 6545 Q-TOF mass spectrometer (Agilent Technologies, Santa Clara, CA, USA) equipped with a Dual AJS ESI source was used. Data were acquired in both positive and negative ion modes. Ion source parameters were a drying gas temperature of 320 °C, drying gas flow of 8 L/min, nebuliser pressure of 35 psi, sheath gas temperature of 350 °C, sheath gas flow of 11 L/min, and capillary voltage of ±3500 V. The scan range was 50–1000 *m*/*z*. MS/MS fragmentation was performed in automatic data-dependent mode, selecting the six most abundant precursor ions for fragmentation with collision energies of 20 and 40 eV. Reference ions were continuously introduced for real-time mass calibration to ensure mass accuracy.

### 2.4. Data Processing and Metabolite Identification

Raw data files from LC-MS and GC-MS were imported into Progenesis (version 3.0, Waters Corporation, Milford, MA, USA) QI or Compound Discoverer 3.3 software (Thermo Fisher Scientific, Waltham, MA, USA), respectively. The processing workflow included peak extraction and deconvolution, retention time correction, and peak alignment. Peak extraction parameters were set with a minimum signal-to-noise ratio of 3 and a mass tolerance of 5 ppm. A final peak area matrix containing all samples and each metabolite feature (defined by exact mass-retention time pair) was generated. Data quality was first assessed by examining the clustering of quality control (QC) samples in total ion current (TIC) plots and principal component analysis (PCA) score plots. Features with a relative standard deviation (RSD) > 30% in QC samples were removed. Subsequently, data were normalised using internal standard correction and total peak area normalisation to correct for instrumental response drift and inter-sample variation. Missing values were imputed using the K-nearest neighbour (KNN) method or half of the minimum value.

According to the Metabolomics Standards Initiative (MSI), the identified metabolites were classified into four confidence levels: Level 1 (confirmed by authentic standards matching retention time and MS/MS), Level 2 (putative identification-high matching scores but no commercial standard), Level 3 (tentative identification-based on accurate mass and molecular formula prediction), and Level 4 (unknown features–no database match). In the present study, only metabolites with MSI Levels 1, 2, and 3 were used for pathway enrichment analysis and biological interpretation. Level 4 unknowns were used exclusively for differential abundance screening and were not included in the biological discussion to avoid overinterpretation. The unexpected compounds lidocaine, scopolamine, lincomycin, avermectin A1a aglycone, gibberellin A53, and xanthoxin are reported as tentative annotations (MSI Level 2/3) or possible exogenous/feed/environment-derived features. Their retention times, *m*/*z* values, MS/MS match scores, database sources, and MSI levels are listed in Supplementary Table S3, and they were not used for pathway enrichment or biological interpretation. Only metabolites with MSI Levels 1–3 were used for pathway enrichment and biological discussion. Level 4 unknowns were used exclusively for differential abundance screening. The specific database versions used were HMDB (version 5.0), METLIN (version 2024), MassBank (version 2024.03, https://massbank.eu/), and mzCloud (version 2024.02, https://www.mzcloud.org/). Precursor mass tolerance was set to ≤5 ppm. For MS/MS matching, a minimum spectral match score of 70% was required for Level 2 (putative identification); for Level 1 confirmation, retention time deviation ≤ 0.2 min and MS/MS match score ≥ 80% were required.

To minimize batch effects, samples were randomized across the analytical run, pooled QC samples were injected after every 10 runs, and the ComBat algorithm (R package “sva” version 3.44.0, R Foundation for Statistical Computing, Vienna,) was applied if residual batch variation was detected.

### 2.5. Multivariate and Univariate Statistical Analysis

Multivariate analysis: Normalised data were imported into SIMCA-P 16.0 software (Sartorius Stedim Data Analytics AB, Umeå, Sweden). Prior to analysis, data were subjected to Pareto scaling (variables centered and divided by the square root of their standard deviation). Unsupervised principal component analysis (PCA) was first performed to visualise overall trends and detect outliers. For the three-group comparison (H vs. L vs. C), partial least-squares discriminant analysis (PLS-DA) was used to maximise inter-group separation. For pairwise comparisons (e.g., H vs. L, H vs. C, C vs. L), orthogonal partial least-squares discriminant analysis (OPLS-DA) was additionally conducted to compute VIP values.

Multivariate models were validated in detail. For PLS-DA, R^2^X (cum), R^2^Y (cum), Q^2^ (cum), and the number of latent variables were recorded. For OPLS-DA, R^2^X (cum), R^2^Y (cum), Q^2^ (cum), and VIP values were recorded. Model validity was assessed by 200 random permutations, and the R^2^ and Q^2^ intercepts and permutation *p* values are reported in Table 1. Cross-validation used [7-fold] cross-validation. Overfitting was assessed by comparing the original Q^2^ with the permuted Q^2^ distribution. Because Q^2^ can be inflated by high dimensionality and large between-group differences, PLS-DA was used mainly for visualization; differential metabolites were defined by OPLS-DA VIP, |log_2_FC|, and FDR-adjusted univariate statistics, not by PLS-DA alone. Differential metabolites were defined using one predefined threshold for all comparisons: OPLS-DA VIP > 1.0, |log_2_FC| > 1.0, and Benjamini–Hochberg-adjusted FDR < 0.05. Nominal *p* values are shown in volcano plots only for visualization. All comparisons follow the same threshold.

## 3. Results and Analysis

### 3.1. Overall Characteristics of the Gut Content Metabolome

**Figure 1:**
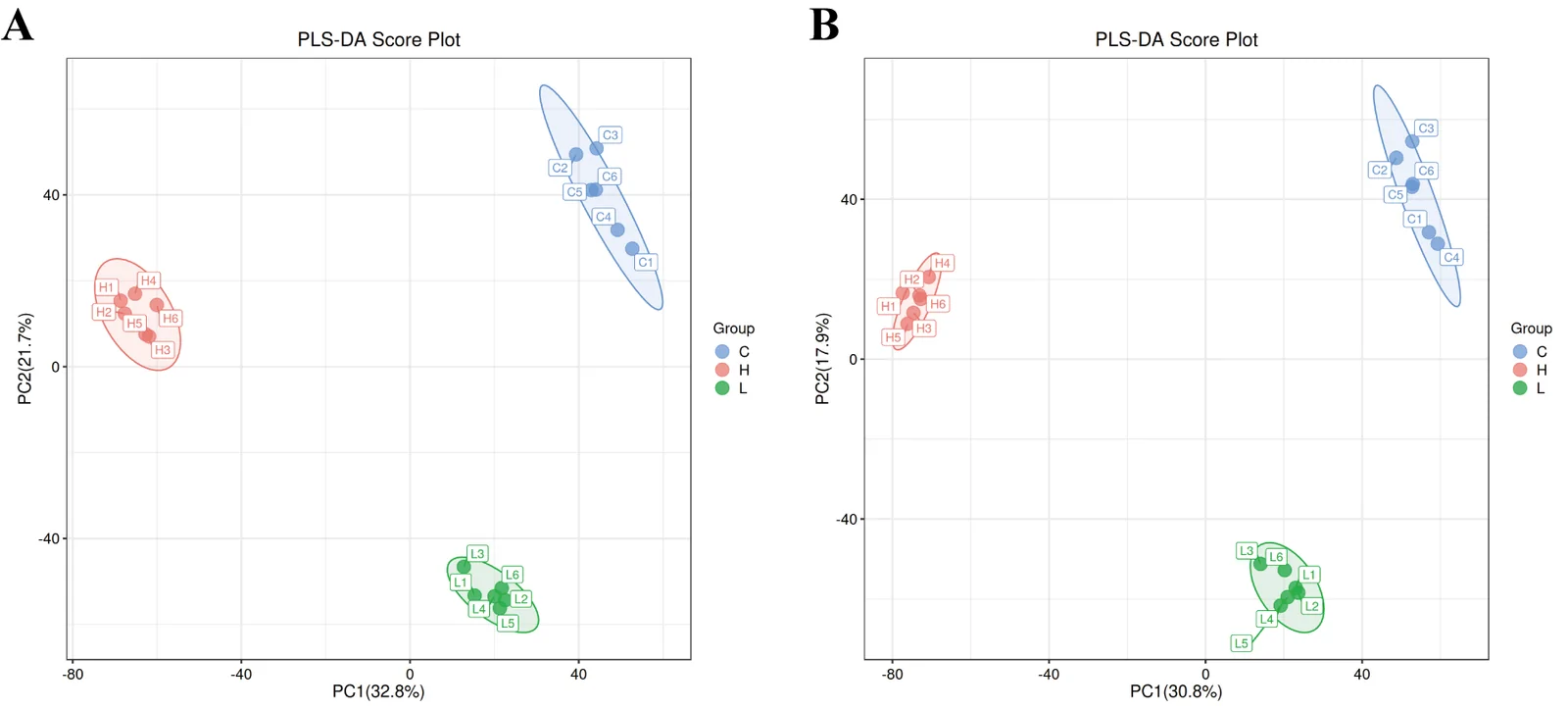
PLS-DA score plots of intestinal contents. (**A**) PLS-DA in positive ion mode; (**B**) PLS-DA in negative ion mode. PLS-DA, partial least-squares discriminant analysis; PC1 and PC2, principal components 1 and 2; ellipses represent 95% Hotelling’s T^2^ confidence intervals. HY, high-altitude yak; LY, low-altitude yak; PC, plateau yellow cattle.

Species-specific features of the gut content metabolic profiles of high-altitude yaks (HYs, hereafter referred to as Group H), low-altitude yaks (LYs, Group L), and plateau yellow cattle (PC, Group C) were analysed using partial least-squares discriminant analysis (PLS-DA) in both positive and negative ion modes. In the positive ion mode, the PLS-DA score plot showed that PC1 and PC2 explained 30.8% and 17.9% of the inter-group variation, respectively, with a cumulative explanation rate of 48.7%. The three groups exhibited completely discrete distribution patterns: Group H was significantly clustered in the negative quadrant of PC1 near zero on PC2; Group L was concentrated in the positive quadrant of PC1 with low PC2 values; and Group C was enriched in the positive quadrant of PC1 with high PC2 values. All samples were tightly enclosed by the 95% Hotelling’s T^2^ confidence ellipses, with no overlap between ellipses, indicating good intra-group biological replicate consistency (Figure 1A). The goodness-of-fit of the PLS-DA model was evaluated (Table 1). For the three-group comparison (H vs. L vs. C), the R^2^Y (cum) was 0.998 and Q^2^ (cum) was 0.990; both values were close to 1, indicating a high model fit. In the negative ion mode, PC1 and PC2 explained 32.8% and 21.7% of the inter-group variation, respectively, raising the cumulative explanation rate to 54.5%. The inter-group separation pattern was highly consistent with that observed in the positive ion mode; samples from Groups H, L, and C still showed non-overlapping discrete distributions, with high intra-group clustering (Figure 1B). Complete PLS-DA and OPLS-DA validation statistics, including latent variables, 200-permutation R^2^/Q^2^ intercepts, and permutation *p* values, are provided in Supplementary Table S2. Model validation parameters further showed (Table 1) that for the three-group comparison, R^2^Y (cum) was 0.997 and Q^2^ (cum) was 0.992, again confirming the reliability of the model. The PLS-DA validation parameters for the three-group comparison are shown in Table 1, and the complete PLS-DA and OPLS-DA validation parameters for all comparisons are provided in Supplementary Table S2. The high consistency of the PLS-DA results between the two ion modes, together with the excellent model validation parameters, demonstrates significant species-specific and altitude-adaptive differentiation in the intestinal metabolite profiles of high-altitude yaks, low-altitude yaks, and plateau yellow cattle. To visualise global metabolic differences among the three groups, we performed PLS-DA (Figure 1). For pairwise comparisons, OPLS-DA was further applied to calculate VIP values (see Methods).

### 3.2. Screening of Differential Metabolites

**Figure 2:**
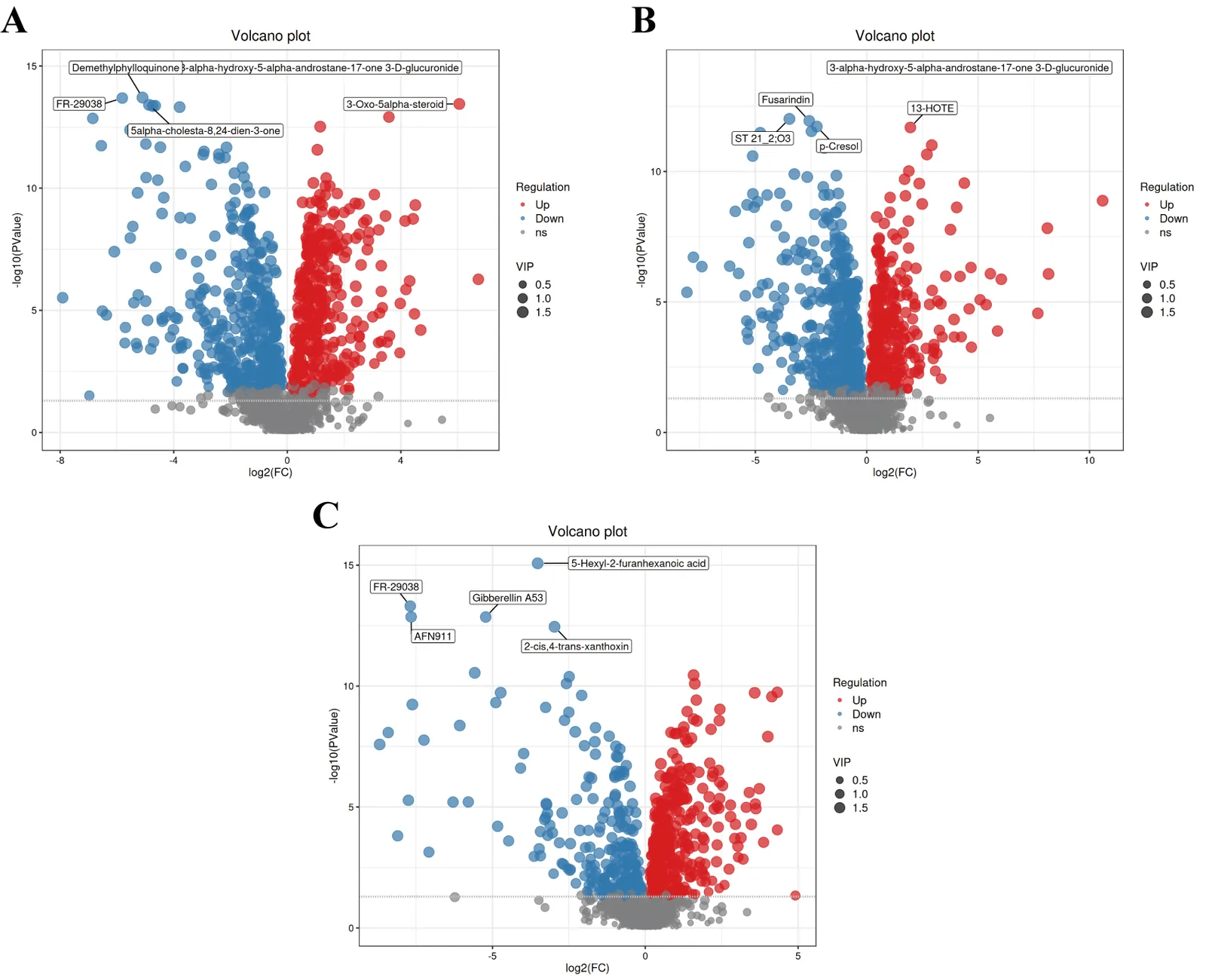
Volcano plots of differential metabolites. (Figure legend: Red dots represent significantly up-regulated metabolites, blue dots represent significantly down-regulated metabolites, and grey dots indicate non-significant differences) (**A**) Volcano plot of differential metabolites between high-altitude yaks and low-altitude yaks; (**B**) Volcano plot of differential metabolites between high-altitude yaks and plateau yellow cattle; (**C**) Volcano plot of differential metabolites between plateau yellow cattle and low-altitude yaks. Note: The volcano plots display −log_10_(nominal *p*-values) for visualization purposes; the significance threshold was set at Benjamini–Hochberg-adjusted FDR < 0.05.

Differential metabolites were screened using the unified criteria described in Section 2.5 (OPLS-DA VIP > 1.0, |log_2_FC| > 1.0, and Benjamini–Hochberg-adjusted FDR < 0.05). The concrete log2 fold changes, *p* values, FDR-adjusted *p* values, regulation directions, and MSI confidence levels for the differentially abundant metabolites are listed in Supplementary Table S3. Volcano plots were used to systematically analyze the differential expression profiles of intestinal metabolomes. In the comparison between high-altitude yaks and low-altitude yaks (Figure 2A), upregulated and downregulated differential metabolites in high-altitude yaks showed a highly enriched symmetric distribution on both sides of the log_2_(FC) axis, and most points exhibited −log_10_(*p* value) > 2 (i.e., *p* < 0.01), indicating highly statistically significant differences. Among them, 3-Oxo-5alpha-steroid was significantly upregulated in high-altitude yaks, whereas Demethylphylloquinone and 5alpha-cholesta-8,24-dien-3-one were significantly downregulated. In addition, Unannotated metabolites (MSI Level 4, e.g., FR-29038 and AFN911) were labeled as statistically significant features; however, they were not used for pathway analysis or biological interpretation. Their potential as altitude-adaptive biomarkers requires further structural identification.

In the comparison between high-altitude yaks and plateau yellow cattle (Figure 2B), differential metabolites also showed a distinct bidirectional regulatory pattern. 13-HOTE was significantly upregulated in high-altitude yaks, and some points showed |log_2_(FC)| > 5, indicating large fold changes. p-Cresol, Fusarindin, and ST 21_2;3 were significantly downregulated in high-altitude yaks.

In the comparison between plateau yellow cattle and low-altitude yaks (Figure 2C), the distribution pattern of differential metabolites was similar to the previous two groups. Notably, 5-Hexyl-2-furanhexanoic acid in plateau yellow cattle showed −log_10_(*p* value) > 15, suggesting extremely high significance. Gibberellin A53 and 2-cis,4-trans-xanthoxin were also significantly downregulated. Moreover, the unannotated metabolites AFN911 and FR-29038, which were downregulated in plateau yellow cattle, displayed high VIP values.

In summary, all three pairwise comparisons exhibited significant regulatory patterns of metabolites, and most differential metabolites showed high statistical significance (*p* < 0.01). All subsequently reported differential metabolites satisfy FDR < 0.05, ensuring that the false positive rate of statistical inference is controlled below 5%. Unannotated metabolites such as FR-29038 and AFN911 deserve focused investigation in subsequent studies.

### 3.3. Overlap and Expression Patterns of Differential Metabolites

**Figure 3:**
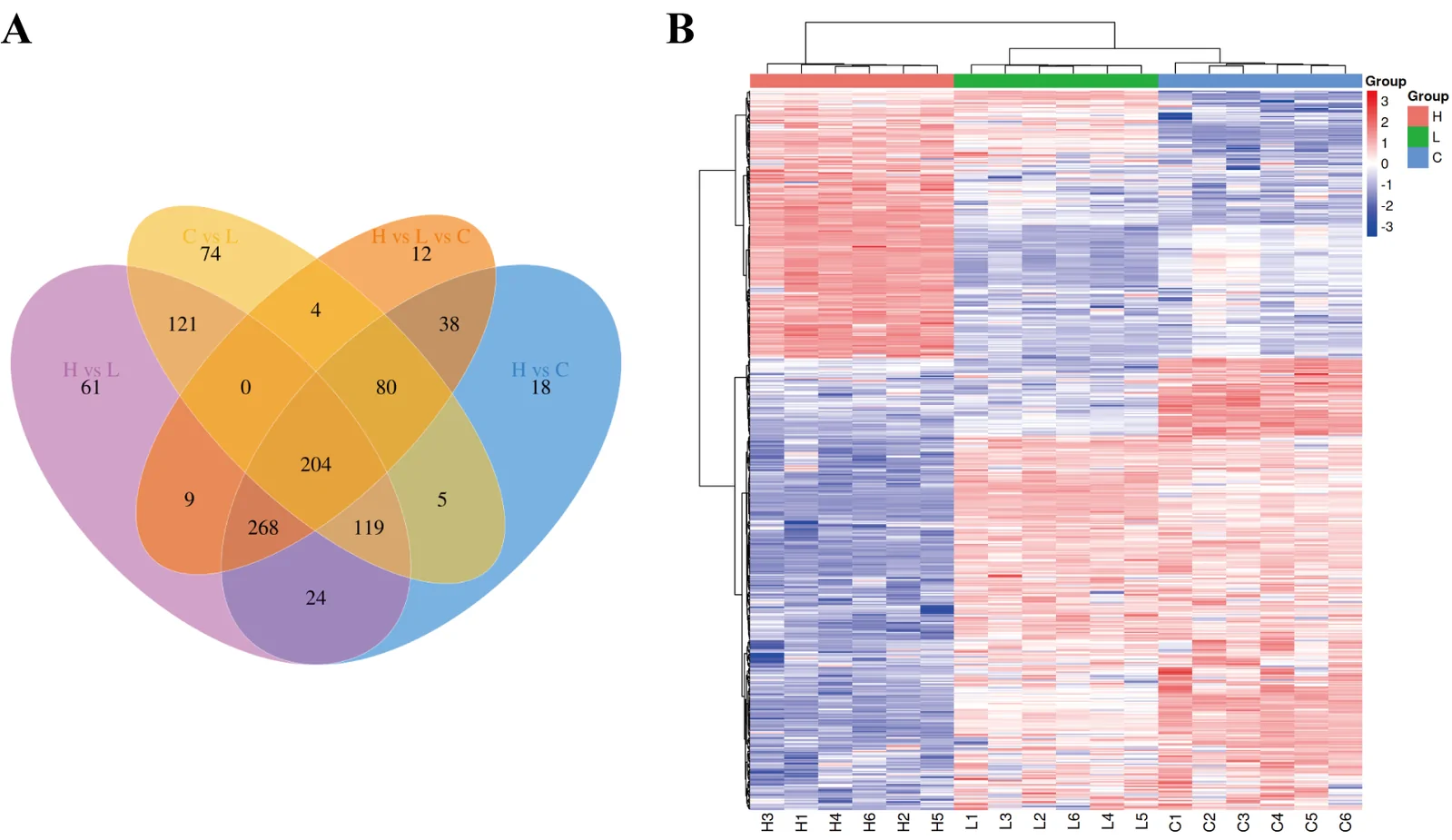
Venn diagram and hierarchical clustering heatmap of differential metabolites. (**A**) Venn diagram showing unique and shared differential metabolites among comparisons. (**B**) Heatmap showing scaled relative abundance of differential metabolites. HY, high-altitude yak; LY, low-altitude yak; PC, plateau yellow cattle. Color scale indicates row-scaled abundance.

Venn analysis was performed to compare overlapping and unique differential metabolites among groups (Figure 3A). In total, 61 differential metabolites were unique to H vs. L, 74 unique to C vs. L, and 18 unique to H vs. C. Twelve unique differential metabolites were identified in the three-group comparison (H vs. L vs. C). Among them, unannotated Level 4 features were excluded from functional enrichment analysis, which were significant only in the overall comparison but not enriched in any pairwise comparison, representing common metabolic differences among the three groups. Two-way clustering was conducted for all samples and differential metabolites, and a clustering heatmap was generated (Figure 3B). Sample clustering revealed distinct intragroup aggregation and intergroup separation among high-altitude yaks, low-altitude yaks, and plateau yellow cattle, indicating obvious species-specific and altitude-adaptive divergence in intestinal metabolic profiles. All differential metabolites were divided into two major clusters: Cluster 1 was highly expressed in high-altitude yaks, lowly expressed in plateau yellow cattle, and moderately expressed in low-altitude yaks. Cluster 2 was highly expressed in plateau yellow cattle, lowly expressed in high-altitude yaks, and also moderately expressed in low-altitude yaks.

In conclusion, intestinal metabolic profiles showed consistent intergroup divergence and intragroup uniformity among high-altitude yaks, low-altitude yaks, and plateau yellow cattle. These differential metabolites displayed a bidirectional expression pattern, and the intermediate expression in the L group reflected a transition between high-altitude adaptation and species-specific regulation.

### 3.4. Group-Specific Distribution of Differential Metabolites

Z-score normalization was performed on core differential metabolites, and scatter plots were constructed (Figure S1). In the high-altitude yak group, multiple differential metabolites showed high Z-scores, including steroid metabolism-related 5alpha-cholesta-8,24-dien-3-one, 5alpha-Androst-1-en-3-one, 3-alpha-hydroxy-5-alpha-androstane-17-one 3-D-glucuronide; vitamin metabolism-related Demethylphylloquinone; host-microbe interaction-related p-Cresol; inflammation and energy metabolism-related Prostaglandin E2, Lovastatin acid; neural and stress regulation-related Lidocaine, Scopolamine; oxidative stress defense-related Uric acid; as well as Berberapten, ST 21_2;O3, Avermectin A1a aglycone, Corosolic acid, ST 24_2;O4, Bufalin, and Lincomycin. The overall metabolic profile suggests that high-altitude yaks are under strong physiological stress and adaptive remodeling.

Highly expressed metabolites in low-altitude yaks were mainly enriched in basal metabolism and antioxidant defense pathways, including the vitamin C biosynthesis precursor L-galactono-1,4-lactone; purine metabolite Allantoin; carbohydrate metabolism-related Fructose; amino acid metabolism-related Gamma-glutamyl-leucine, L-Tryptophan, N-Malonyltryptophan; nucleic acid metabolism-related Deoxyguanosine, Vidarabine; oxidative stress regulation-related 2,4-Quinolinediol; and D-Pinitol, Cinnamic acid, (S)-2-Methylbutanal, 3-(Carboxymethylamino)propanoic acid, Acetylpyruvate, P-Coumaraldehyde, D-Phenyllactic acid, Tyramine, (Z)-4-(1-Propenyl)phenol, 7′,8′-Dihydro-8′-hydroxyreticulataxanthin, and 5-Hydroxy-N-formylkynurenine. The metabolic status of low-altitude yaks was more oriented toward homeostasis, basal anabolism, and systemic redox balance.

Highly expressed metabolites in plateau yellow cattle included lipid metabolism-related 5-Hexyl-2-furanhexanoic acid; connective tissue protein Elastin; sulfur-containing amino acid metabolite Cysteine S-sulfate; fungal-derived Fusarindin; coenzyme Q family member Coenzyme Q9; anti-inflammatory Quinacrine; carbohydrate metabolism-related D-Ribulose 5-phosphate; as well as 3-Epidemissoin, Lithocholate 3-O-glucuronide, FO 2546M, 4-Sulfobenzyl alcohol, Dioxinoacrimarine A, 8(R)-Hydroperoxyoctadecanoic acid, N2-Succinoylarginine, 5-[(4-Hydroxyphenyl)ethenyl]butenyl-1,3-benzenediol, and (S)-beta-macrocapene, demonstrating clear species specificity.

**Figure 4:**
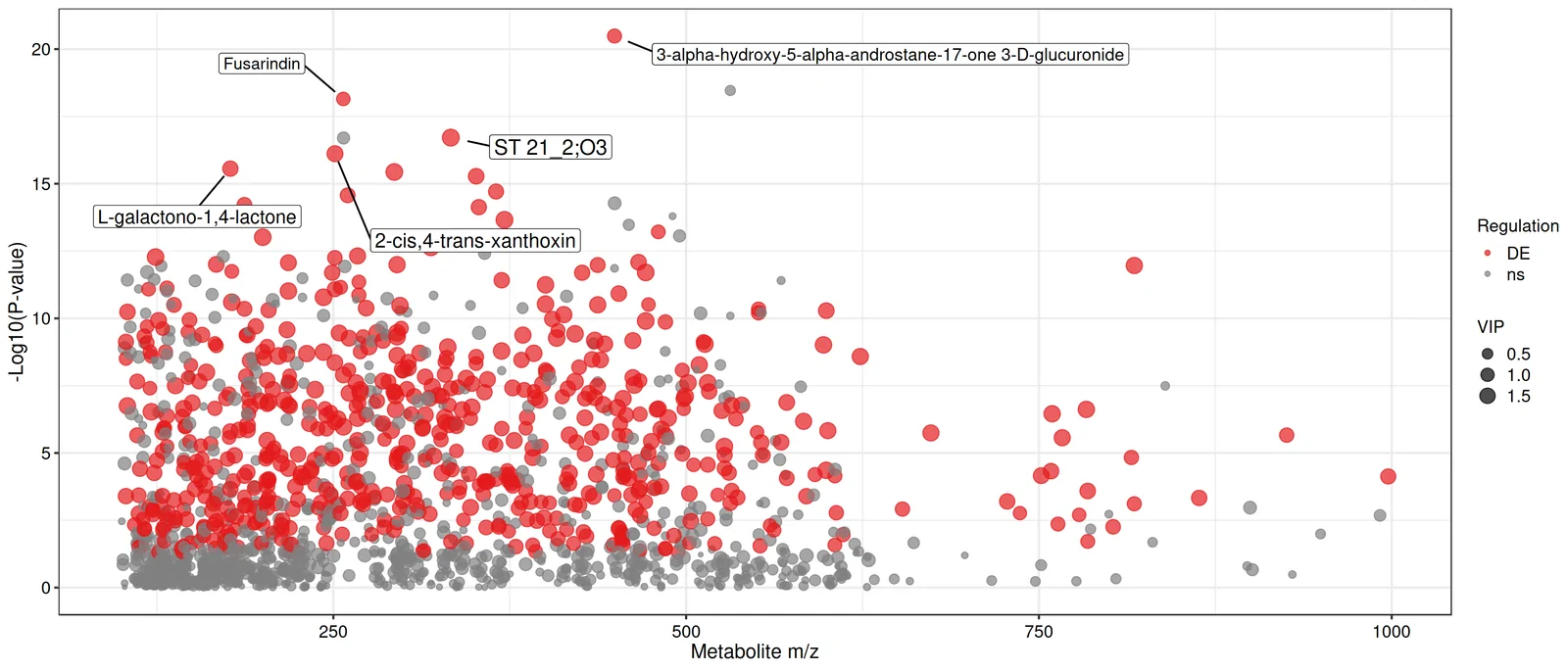
Scatter plot of core differential metabolites based on metabolite *m*/*z* and −log_10_(*p* value). Red dots (DE) indicate significantly differential metabolites; grey dots (ns) indicate non-significant metabolites. Dot size indicates VIP score (0.5, 1.0, 1.5). *m*/*z*, mass-to-charge ratio; VIP, variable importance in projection; DE, differential; ns, not significant.

Differential scatter plots were constructed based on the *m*/*z* and *p* values of metabolites (Figure 4). Significantly differential metabolites were widely distributed within 0–1000 *m*/*z*, and most red points showed −log_10_(*p* value) > 5 (*p* < 10^−5^), indicating very high statistical significance. Points with high VIP values were mainly concentrated in 0–500 *m*/*z*, suggesting that low-molecular-weight metabolites contributed more to intergroup separation. Nonsignificant metabolites were mainly distributed at −log_10_(*p* value) < 2.5, indicating that most metabolites differed significantly among the three groups.

Key metabolites were highlighted as follows: 3-alpha-hydroxy-5-alpha-androstane-17-one 3-D-glucuronide: located at 0–500 *m*/*z*, −log_10_(*p* value) > 20, high VIP, a steroid metabolite. Fusarindin: located at 250–500 *m*/*z*, −log_10_(*p* value) > 17, high VIP, a fungal-derived metabolite. ST 21_2;O3: located at 250–500 *m*/*z*, −log_10_(*p* value) > 15, high VIP, an unannotated metabolite. 2-cis,4-trans-xanthoxin: located at 250–500 *m*/*z*, −log_10_ (*p* value) > 15, high VIP, a carotenoid metabolite. L-galactono-1,4-lactone: located at 0–250 *m*/*z*, −log_10_ (*p* value) > 15, high VIP, a vitamin C biosynthesis precursor.

### 3.5. Enriched Metabolic Pathways of Differential Metabolites

**Figure 5:**
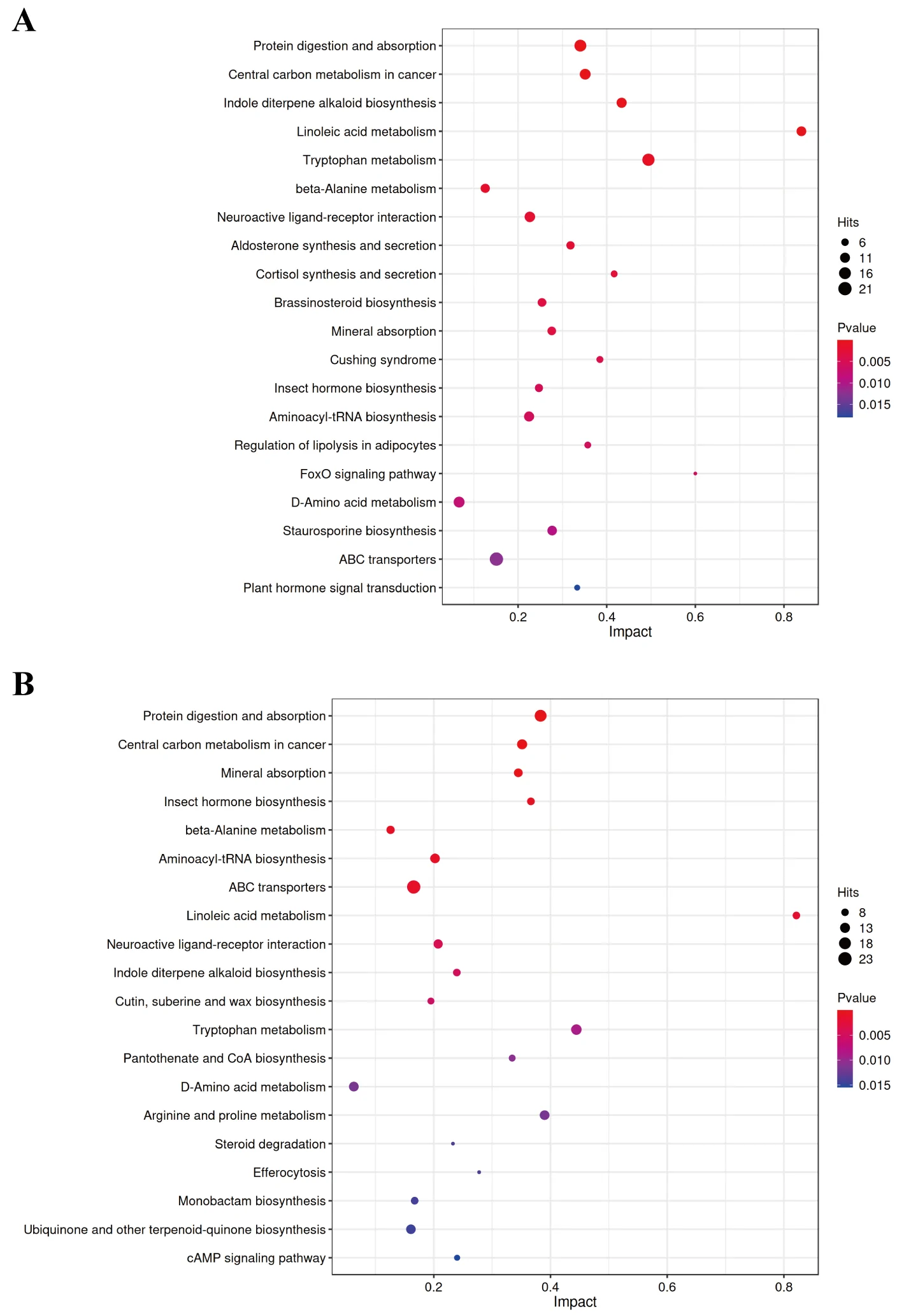
KEGG pathway enrichment of differential metabolites. (**A**) H vs. L; (**B**) H vs. C. Hits, number of differential metabolites mapped to the pathway; Impact, pathway impact value; P, enrichment *p* value. KEGG, Kyoto Encyclopedia of Genes and Genomes.

Pathway enrichment was performed using MetaboAnalyst 5.0 (https://www.metaboanalyst.ca/) against the KEGG (https://www.kegg.jp/) Bos taurus pathway metabolite set. The numbers of input metabolites for H vs. L, H vs. C, C vs. L, and H vs. L vs. C were analysed, respectively. Enrichment significance was assessed by Fisher’s exact test, and multiple testing was corrected using the Benjamini–Hochberg FDR. Pathway topology was evaluated using the Impact value. Only MSI Level 1–3 metabolites were included; Level 4 unknowns were excluded from pathway analysis. Functional pathway enrichment analysis was performed only on differential metabolites with MSI confidence Levels 1, 2, or 3 (i.e., excluding unannotated Level 4 features) from intestinal contents of high-altitude yaks, low-altitude yaks, and plateau yellow cattle. Visualization based on Impact value, *p* value, and number of hits is shown below. In the H vs. L comparison (Figure 5A), linoleic acid metabolism showed the highest Impact value, representing the most discriminative pathway between groups. Meanwhile, tryptophan metabolism and protein digestion and absorption were also significantly enriched. In addition, indole diterpene alkaloid biosynthesis, central carbon metabolism in cancer, beta-alanine metabolism, and neuroactive ligand–receptor interaction exhibited significant enrichment. Pathways such as central carbon metabolism in cancer, prostate cancer, insect hormone biosynthesis, and axon regeneration are named according to KEGG maps and arise from computational mapping of differential metabolites to KEGG metabolite sets. They do not imply that yaks or cattle undergo cancer, insect hormone biosynthesis, or axon regeneration; they are listed for completeness and are not interpreted as direct biological processes. The complete KEGG pathway enrichment statistics, including group, pathway ID, pathway name, total metabolites, *p* values, and FDR values, are provided in Supplementary Table S4.

In the H vs. C comparison (Figure 5B), linoleic acid metabolism remained the most significantly different pathway. Protein digestion and absorption, ABC transporters, tryptophan metabolism, beta-alanine metabolism, and aminoacyl-tRNA biosynthesis were significantly enriched, while neuroactive ligand–receptor interaction and indole diterpene alkaloid biosynthesis also showed group-specific metabolic differences. In the C vs. L comparison (Figure S2), pyrimidine metabolism was the most representative differential pathway. Protein digestion and absorption, ABC transporters, beta-alanine metabolism, D-amino acid metabolism, and mineral absorption were significantly enriched. Additionally, alanine, aspartate and glutamate metabolism and histidine metabolism showed obvious intergroup differences.

In the three-group comparison (H vs. L vs. C; Figure S3), linoleic acid metabolism was again the pathway with the strongest biological effect underlying intergroup metabolic differences. The ABC transporters pathway had the largest number of hits, with an Impact value of approximately 0.15 and *p* < 0.01, indicating its involvement in the transport of numerous differential metabolites. Meanwhile, protein digestion and absorption, indole diterpene alkaloid biosynthesis, cutin, suberine and wax biosynthesis, neuroactive ligand–receptor interaction, and beta-alanine metabolism were also significantly enriched. Furthermore, tryptophan metabolism, insect hormone biosynthesis, and axon regeneration showed moderate enrichment. Pathways such as cAMP signaling pathway, steroid degradation, prostate cancer, and FoxO signaling pathway were involved in intergroup differences but with relatively weak biological effects.

### 3.6. Analysis of Core Pathways Related to Altitude Adaptation

Statistical analyses of differential metabolites within linoleic acid metabolism and pyrimidine metabolism, which were significantly enriched across comparisons, were performed (Table 2). In the H vs. L group, linoleic acid metabolism contained 10 DAMs, covering three major categories: polyunsaturated fatty acids (arachidonic acid, linoleic acid, dihomo-gamma-linolenic acid, gamma-linolenic acid), hydroperoxide derivatives (13-L-Hydroperoxylinoleic acid, 8(R)-Hydroperoxylinoleic acid, 10R-HpODE), and oxidative cleavage products (3-HODE + 9-HODE, FA 18:2; O, 9,10-DiHOME). In the H vs. C group, linoleic acid metabolism included 8 DAMs, seven of which (linoleic acid, dihomo-gamma-linolenic acid, 3-HODE + 9-HODE, FA 18:2; O, 9,10-DiHOME, 8(R)-Hydroperoxylinoleic acid, 10R-HpODE) were shared with H vs. L, plus the phospholipid metabolite PC (16:0/18:2 (9Z, 12Z)). In the C vs. L group, pyrimidine metabolism was enriched with 12 DAMs covering the entire process of pyrimidine nucleotide metabolism, including synthetic precursors (L-glutamine, L-dihydroorotic acid, ureidosuccinic acid), pyrimidine bases (uracil, thymine, 5,6-dihydrouracil), nucleotides (uridine 5′-monophosphate, thymidine-5′-monophosphoric acid), and nucleosides (uridine, cytidine, deoxycytidine, thymidine). In the H vs. L vs. C group, seven DAMs in the linoleic acid metabolism pathway were shared key metabolites of H vs. L and H vs. C, including PC (16:0/18:2 (9Z, 12Z)), linoleic acid, dihomo-gamma-linolenic acid, 3-HODE + 9-HODE, 9,10-DiHOME, 8(R)-Hydroperoxylinoleic acid, and 10R-HpODE. Arachidonic acid, gamma-linolenic acid, and 13-L-Hydroperoxylinoleic acid were unique to H vs. L.

In summary, core differential metabolites of linoleic acid metabolism are highly conserved among high-altitude yaks, low-altitude yaks, and plateau yellow cattle, representing a candidate shared enrichment pattern of high-altitude hypoxia adaptation in Bovidae. The unique metabolites in H vs. L and the specific enrichment of pyrimidine metabolism in C vs. L reflect species-specific adaptive characteristics.

## 4. Discussion

High-altitude environments are characterized by hypoxia, low temperature, and limited nutrient availability, yet yaks exhibit strong adaptability to these harsh conditions. The gut serves as a critical interface for host-microbe interactions, and its metabolic profile can directly reflect the adaptive changes in the host in response to external environments. In this study, untargeted metabolomics was employed to characterize intestinal metabolites in high-altitude yaks (Group H), low-altitude yaks (Group L), and plateau yellow cattle (Group C). We elucidated the adaptive differentiation of intestinal metabolism and identified key differential metabolites and core metabolic pathways associated with high-altitude adaptation, providing novel theoretical insights for further exploring the mechanisms underlying high-altitude adaptation in yaks.

The overall characteristics of the metabolome reflect the combined effects of host genetic background, physiological status, and environmental factors [12]. In the present study, PLS-DA analyses in both positive and negative ion modes revealed distinct separation among the three groups, indicating that the samples were representative and reliable. PLS-DA model validation parameters (R^2^Y (cum) > 0.997, Q^2^ (cum) > 0.990) further confirmed the accuracy and credibility of the metabolic differentiation results. Yaks and cattle belong to different genera within the Bovidae family and exhibit substantial genetic divergence. In contrast, low-altitude yaks, which have been reared under long-term low-altitude conditions, displayed partially attenuated high-altitude adaptive traits. This finding is highly consistent with studies on other highland-dwelling species, supporting that high-altitude adaptation arises from the interplay between genetics and environment. For instance, plateau frogs employ tissue-specific metabolic suppression and regulatory strategies to systematically cope with hypoxic stress [13]. Genetically adapted “highland mice” demonstrate that specific genetic variations play a decisive role in maintaining metabolic homeostasis [14]. Moreover, when high-altitude human populations return to lowland areas, their plasma and fecal metabolomes undergo significant perturbations linked to “de-adaptation” syndromes, providing inverse evidence that environmental reversal weakens adaptive metabolic signatures.

Collectively, multiple cross-species investigations demonstrate that the host metabolome is shaped by both genetic background and environmental cues. Accordingly, we selected high-altitude yaks (Group H), low-altitude yaks (Group L), and plateau yellow cattle (Group C) as study subjects. Hypothesis-generating comparative framework—altitudinal environment and species genetics—represents an optimal comparative framework for investigating high-altitude adaptation. Intestinal metabolites in yaks exhibited a significant bidirectional regulatory pattern among the experimental groups, with the majority of metabolites showing *p* < 0.01, indicating adaptive remodeling of metabolic networks during species divergence and high-altitude adaptation. This mode of metabolic regulation is consistent with environmental adaptation studies in other animals, although direct causal evidence in yaks is still lacking. For example, mice harboring specific high-altitude genetic mutations precisely bidirectionally modulate glucose, fatty acids, and other metabolites under hypoxic stress to maintain metabolic homeostasis rather than adopting unidirectional metabolic bias [14]. Similarly, during cold adaptation in ruminants, cold-tolerant sheep upregulate propionate and butyrate production via gut microbiota for efficient energy supply. In contrast, cold-sensitive sheep rely more on acetate production and energy-consuming thermogenesis, displaying divergent energy utilization strategies [15]. Collectively, bidirectional regulation of differential metabolites represents a core adaptive strategy employed by animals in response to environmental stress.

In the comparison between high-altitude yaks and low-altitude yaks, 3-Oxo-5α-steroid was significantly upregulated in high-altitude yaks, whereas Demethylphylloquinone and 5α-cholesta-8,24-dien-3-one were significantly downregulated. Steroid metabolites play vital roles in regulating host energy metabolism, stress responses [16], and immune function [17]. As an intermediate in steroid metabolism, 3-Oxo-5α-steroid is critically involved in diverse physiological and pathological processes. Its marked upregulation in high-altitude yaks may constitute an important metabolic adaptation to hypoxic stress at high elevations. Demethylphylloquinone is a naturally occurring form of vitamin K that participates in blood coagulation and bone metabolism. Its significant downregulation in high-altitude yaks may be attributed to nutritional differences in forage. The scarcity of nutrients in high-altitude pastures limits dietary vitamin K1 intake, leading to reduced endogenous levels. 5α-cholesta-8,24-dien-3-one is a cholesterol derivative involved in bile acid and steroid hormone synthesis. Its downregulation suggests that high-altitude yaks adjust cholesterol metabolism to meet altered energy demands under high-altitude conditions. In the comparison between high-altitude yaks and plateau yellow cattle, the differences observed between HYs and PC likely arise from a combination of species-specific genetic background and the modest altitude difference (300 m); therefore, these results should not be interpreted solely as altitude adaptation.

13-HOTE was significantly upregulated in high-altitude yaks, while p-Cresol, Fusarindin, and ST 21_2; 3 were significantly downregulated. 13-HOTE possesses anti-inflammatory and antioxidant activities and participates in host immune regulation and oxidative stress modulation [18]. Its pronounced upregulation in high-altitude yaks likely reflects severe oxidative stress under high-altitude hypoxia. By specifically elevating 13-HOTE levels, the host enhances systemic and local antioxidant defense to alleviate oxidative damage. This inference is indirectly supported by studies in other animal models. For instance, recent research confirmed that α-linolenic acid and its oxidized lipids (including 13-HOTrE) exert clear anti-inflammatory and antioxidant effects by activating PPARγ, inhibiting proinflammatory pathways such as NF-κB, and upregulating antioxidant enzymes [19]. Another study reported that 12/15-lipoxygenase suppresses inflammation and reduces oxidative stress during wound repair in mice by generating PPARγ-activating oxidized lipids such as 13-HOTrE [20]. These findings align with our hypothesis and collectively suggest a protective role of 13-HOTE in mitigating oxidative stress.

p-Cresol was significantly downregulated in the intestinal contents of high-altitude yaks [21]. Its production can be influenced by specific gut microbiota, including Clostridiaceae, and has been associated with gut microbial structure and function [22,23]. However, because no microbiome analysis was performed in this study, this interpretation remains hypothetical and requires future metagenomic or microbiome validation. The downregulation of p-cresol may be associated with, but is not proven to be, a metabolic adaptation in high-altitude yaks. In the comparison between plateau yellow cattle and low-altitude yaks, 5-Hexyl-2-furanhexanoic acid showed extremely significant differences in plateau yellow cattle; 2-cis,4-trans-Xanthoxin and gibberellin A53 were tentatively annotated and may originate from plant/feed material. Therefore, their biological interpretation in the host should be treated with caution. If confirmed by authentic standards, reduced xanthoxin might indicate attenuated abscisic acid-dependent stress signaling, and reduced gibberellin A53 might indicate suppressed growth-promoting signaling; however, these remain hypotheses requiring validation and were not used to support the core conclusion. 5-Hexyl-2-furanhexanoic acid is a potent endogenous antioxidant and lipid metabolism regulator that scavenges reactive oxygen species (ROS) and maintains cell membrane stability under hypoxic stress [24]. Yaks resist hypoxia-induced oxidative damage via reprogrammed lipid metabolism and enhanced antioxidant defense [25]. The marked divergence of this furan fatty acid may represent a species-specific adaptive strategy in plateau yellow cattle against high-altitude oxidative stress.

In contrast, Gibberellin A53 and 2-cis,4-trans-xanthoxin were significantly decreased in plateau yellow cattle. As a direct biosynthetic precursor of abscisic acid, reduced 2-cis,4-trans-xanthoxin indicates attenuated activity of abscisic acid-dependent stress signaling [26], suggesting that plateau yellow cattle have transitioned from acute hypoxic stress to stable physiological homeostasis. This is consistent with multi-omics studies showing that high-altitude adapted ruminants maintain mild and sustained stress signaling to avoid excessive energy consumption under chronic hypoxia [27]. Meanwhile, Gibberellin A53, a key driver of cell proliferation, organismal growth, and energy-costly anabolism [28], was markedly reduced in plateau yellow cattle, indicating targeted suppression of growth-promoting signaling. Experiments in yaks and sheep consistently confirm that downregulation of Gibberellin A53-related pathways is a conserved energy adaptive strategy in high-altitude ruminants [25], which curtails energy expenditure associated with growth and redirects metabolism toward homeostatic adaptation via energy reprogramming [27]. In summary, metabolic comparisons between plateau yellow cattle and low-altitude yaks revealed extremely significant differences in 5-Hexyl-2-furanhexanoic acid and significant downregulation of Gibberellin A53 and 2-cis,4-trans-xanthoxin(tentative annotation, MSI Level 2/3). The former supports species-specific hypoxia adaptation via antioxidant defense and lipid reprogramming, whereas downregulation of the latter two weakens stress signaling and reduces growth-related energy consumption, representing a conserved mechanism regulating the transition from acute stress to homeostatic adaptation in high-altitude animals. These features may be exogenous, feed-derived, environmental, or medication-derived. They were treated as tentative annotations and were not used for pathway enrichment or biological interpretation.

Venn diagram analysis in this study showed that the 61 unique differential metabolites in H vs. L mainly reflect adaptive differences between yaks at different altitudes, the 74 unique metabolites in C vs. L correspond to species-specific divergence between plateau yellow cattle and low-altitude yaks, and the 12 unique metabolites in H vs. L vs. C may represent candidate shared metabolic features shaped by both genetics and high-altitude environment. Notably, the unannotated metabolites FR-29038 and AFN911 identified in this study exhibited high VIP values and significant differences, suggesting they may represent novel small molecules associated with high-altitude adaptation and serve as candidate biomarkers for future investigation.

Functional pathway enrichment analysis revealed that significantly enriched pathways across comparison groups were closely related to energy metabolism, stress responses, nutrient absorption, and other physiological processes. Linoleic acid metabolism was significantly enriched in all comparisons (H vs. L, H vs. C, H vs. L vs. C) with highly conserved core differential metabolites, indicating that linoleic acid metabolism is a consistently enriched candidate pathway with a conserved enrichment pattern across comparisons underlying yak adaptation to high altitude. However, due to the sensitivity of pathway enrichment to the input metabolite list and the incompleteness of the KEGG database for non-model species, this conclusion should be further validated in independent cohorts. However, the phospholipid metabolite PC (16:0/18:2 (9Z, 12Z)) was uniquely detected in H vs. C, suggesting that phospholipid remodeling participates in hypoxia adaptation mediated by linoleic acid metabolism. Accumulating evidence confirms that yaks adapt to extreme high-altitude environments via lipid metabolic reprogramming. Polyunsaturated fatty acids and their oxidized derivatives effectively alleviate hypoxia-induced oxidative damage and enhance stress resistance [29,30]. Linoleic acid metabolites dominate metabolic regulation following acute hypoxia and are associated with phenotypic differences in hypoxia adaptation [31]. Furthermore, H vs. L specifically showed enrichment of arachidonic acid, γ-linolenic acid, and 13-L-Hydroperoxylinoleic acid. As an important lipid signaling molecule, arachidonic acid metabolism is activated under hypoxia [32], further strengthening the adaptive advantage of yaks relative to plateau yellow cattle. Pyrimidine metabolism, a core pathway for nucleic acid synthesis [33], energy transfer [34], and cell proliferation [35], was significantly enriched only in C vs. L. Twelve differential metabolites covered the full spectrum of de novo pyrimidine synthesis, base conversion, and nucleoside metabolism, suggesting that plateau yellow cattle may achieve hypoxia adaptation partly via pyrimidine reprogramming. Hypoxia significantly perturbs nucleotide homeostasis [36], and pyrimidine reprogramming maintains steady-state DNA replication [37], RNA transcription [34], and energy (ATP/GTP) supply by balancing nucleotide synthesis and degradation [38], representing a critical metabolic adaptation in high-altitude mammals. Collectively, our results reveal that yaks rely on conserved linoleic acid metabolism as a core adaptive strategy, whereas plateau yellow cattle depend on pyrimidine reprogramming to sustain physiological homeostasis under hypoxia.

Beyond linoleic acid and pyrimidine metabolism, other pathways including tryptophan metabolism and ABC transporters were also significantly enriched in different comparisons, jointly mediating species-specific and altitude-adaptive regulation. Differential metabolites in the kidneys of high-altitude yaks are significantly enriched in ABC transporter pathways, with the key gene ABCB5 identified [39]. In the intestine, transcriptomic analysis revealed upregulation of the tryptophan metabolic enzymes IDO1 and KYNU, as well as significantly high expression of ABCG2, another ABC transporter involved in transmembrane transport of vitamins and other compounds [40]. These findings imply that the yak kidney and intestine may rely on distinct members of the ABC transporter family to mediate adaptive transport of metabolites and vitamins. Meanwhile, the intestine specifically activates tryptophan metabolism to synergistically cope with high-altitude hypoxia. Tryptophan metabolism and ABC transporters play important roles in hypoxia adaptation and related processes. In rat models of chronic hypoxia-induced pulmonary hypertension, tryptophan metabolism is severely disrupted [41]. Among tryptophan derivatives, 5-methoxytryptophan (5-MTP) is reduced during hypoxic lung injury, and exogenous 5-MTP alleviates acute hypobaric hypoxia-induced lung damage by targeting PRDX6 and reducing lipid peroxidation [42]. A microbiota-metabolism axis links tryptophan metabolic disturbance and gut dysbiosis to chronic hypoxia-induced pulmonary hypertension [41]. Tryptophan supplementation attenuates acute hypoxic intestinal injury by improving gut barrier integrity and microbial homeostasis [43]. The enhanced tryptophan metabolism observed in high-altitude yaks may therefore reflect a compensatory mechanism for high-altitude adaptation. This study identified numerous key differential metabolites and core pathways associated with high-altitude adaptation, especially the unannotated metabolites FR-29038 and AFN911, providing novel candidate targets for exploring potential altitude-adaptive biomarkers.

This study has several limitations. The experimental design cannot fully distinguish the effects of altitude and species: the comparison between HYs and LYs can mainly reflect altitude adaptation, the comparison between HYs and PC primarily indicates genetic differences among species, and the comparison between PC and LYs is affected by both altitude and species, so the results of the latter two comparisons cannot be simply attributed to altitude or species alone. In addition, the sample size of six individuals per group is relatively small. Effect sizes (log_2_FC) and 95% confidence intervals for key differential metabolites are reported in Supplementary Table S3; validation in larger independent cohorts is still recommended. Moreover, unannotated metabolites such as FR-29038 and AFN911 are strictly regarded as MSI Level 4 unknowns and require further structural identification before they can be used in biological interpretation. Despite these limitations, the consistent enrichment of linoleic acid metabolism across multiple comparisons together with good model validation parameters confirms the reliability of the core findings of this study. The KEGG pathway annotation used in this study is based on the bovine (Bos taurus) reference genome. Although yaks and plateau yellow cattle are phylogenetically close to Bos taurus, species-specific metabolites or pathways may be missing, potentially leading to underestimation of certain biological pathways. Due to the limited sample size (n = 6 per group) and the large number of differential metabolites, the pathway enrichment results should be validated in larger cohorts. The conservative enrichment patterns across multiple comparisons (H vs. L, H vs. C, H vs. L vs. C) strengthen the reliability of our main findings.

## 5. Conclusions

This untargeted metabolomic comparison suggests that linoleic acid metabolism is a consistently enriched candidate pathway associated with high-altitude adaptation in yaks, whereas pyrimidine metabolism may be associated with species-specific adaptation in plateau yellow cattle. Tryptophan metabolism and ABC transporters may relate to potential host–microbiome interactions under chronic hypoxia, but microbiome validation is required. These findings provide candidate metabolic signatures for plateau ruminant adaptation, genetic resource conservation, and precision husbandry. However, the design cannot fully decouple altitude from species, diet, and environmental effects; the limited sample size (n = 6 per group), MSI Level 4 unknowns, and Bos taurus-based KEGG annotation should be addressed in larger cohorts, targeted metabolomics, and multi-omics studies.

## Figures and Tables

**Table 1:** PLS-DA model validation parameters.

Comparison (H vs. L vs. C)	R^2^X (cum)	R^2^Y (cum)	Q^2^ (cum)
ESI^+^	0.486	0.998	0.990
ESI^−^	0.546	0.997	0.992

**Table 2:** Key differentially abundant metabolites enriched in linoleic acid metabolism and pyrimidine metabolism pathways. Note: All listed metabolites are MSI Level 1–3 unless otherwise stated. Level 4 unknowns were excluded from pathway enrichment and biological interpretation.

Group	Pathway	Compound_Name
H vs. L_group	Linoleic acid metabolism	Arachidonic acid; Linoleic acid; Dihomo-gamma-linolenic acid; 13-L-Hydroperoxylinoleic acid; Gamma-Linolenic acid; 3-HODE + 9-HODE; FA 18: 2;O; 9,10-DiHOME; 8(R)-Hydroperoxylinoleic acid; 10R-HpODE
H vs. C_group	Linoleic acid metabolism	PC (16:0/18: 2 (9Z, 12Z)); Linoleic acid; Dihomo-gamma-linolenic acid; 3-HODE + 9-HODE; FA 18:2; O; 9,10-DiHOME; 8(R)-Hydroperoxylinoleic acid; 10R-HpODE
C vs. L_group	Pyrimidine metabolism	L-Glutamine; Uridine 5′-monophosphate; Uracil; Thymine; Thymidine; Uridine; L-Dihydroorotic acid; Thymidine 5′-monophosphate; 5,6-Dihydrouracil; Ureidosuccinic acid; Cytidine; Deoxycytidine
H vs. L vs. C group	Linoleic acid metabolism	PC (16:0/18:2 (9Z, 12Z)); Linoleic acid; Dihomo-gamma-linolenic acid; 3-HODE + 9-HODE; 9,10-DiHOME; 8(R)-Hydroperoxylinoleic acid; 10R-HpODE

## Data Availability

The original contributions presented in this study are included in the article/Supplementary Material. Further inquiries can be directed to the corresponding authors.

## Supplementary Materials

The following supporting information can be downloaded at https://www.mdpi.com/article/10.3390/vetsci13101063/s1. Table S1: Animal and sampling metadata. Table S2: Model validation parameters for PLS-DA and OPLS-DA. Table S3: Differentially abundant metabolites with concrete values. Table S4: Enriched pathways with concrete values. Figure S1: Z-score scatter plot showing group-specific expression patterns of core differential metabolites in high-altitude yaks, low-altitude yaks, and plateau yellow cattle. Figure S2: Pathway enrichment analysis of differential metabolites between plateau yellow cattle and low-altitude yaks, with pyrimidine metabolism as the most significantly enriched pathway. Figure S3: Pathway enrichment analysis of differential metabolites across the three groups, highlighting linoleic acid metabolism as the core pathway and ABC transporters as the pathway with the largest number of hits.
