# Supplementary material for: A Systematic Comparison of Differential Metabolites and Functional Pathways in the Gut Contents of Yaks and Plateau Yellow Cattle Across Altitudinal Gradients
Source: Vet Sci. 2026 Oct 9;13(10):1063. doi: 10.3390/vetsci13101063 (PMC13709441; doi:10.3390/vetsci13101063)
Supplement: Supplementary file 1 [file vetsci-13-01063-s001.zip › Supplementary Table S1.pdf]

Supplementary Table S1. Animal and sampling metadata

Group	Species	Altitude (m)	Age / Sex / Physiological status	Body weight (kg)	Sampling location
HY	Yak	3800	4–5 years / Female / Non-pregnant, non-lactating	280 ± 25	Xiahe County, Gannan, Gansu, China
LY	Yak	2500	4–5 years / Female / Non-pregnant, non-lactating	275 ± 22	Xiahe County, Gannan, Gansu, China
PC	Plateau yellow cattle	3500	4–5 years / Female / Non-pregnant, non-lactating	290 ± 28	Xiahe County, Gannan, Gansu, China
