# Supplementary material for: A Systematic Comparison of Differential Metabolites and Functional Pathways in the Gut Contents of Yaks and Plateau Yellow Cattle Across Altitudinal Gradients
Source: Vet Sci. 2026 Oct 9;13(10):1063. doi: 10.3390/vetsci13101063 (PMC13709441; doi:10.3390/vetsci13101063)
Supplement: Supplementary file 1 [file vetsci-13-01063-s001.zip › Supplementary Table S2.pdf]

Supplementary Table S2. Model validation parameters (PLS-DA and OPLS-DA)

Comparison	Model	ESI+ $R^2Y(cum)$	ESI+ $Q^2(cum)$	ESI− $R^2Y(cum)$	ESI− $Q^2(cum)$
H vs L	PLS-DA	1.000	0.982	1.000	0.987
H vs C	PLS-DA	1.000	0.983	1.000	0.990
C vs L	PLS-DA	1.000	0.954	1.000	0.975
H vs L vs C	PLS-DA	0.998	0.990	0.997	0.992
H vs L	OPLS-DA	1.000	0.975	1.000	0.983
H vs C	OPLS-DA	1.000	0.974	1.000	0.980
C vs L	OPLS-DA	1.000	0.926	1.000	0.953
H vs L vs C	OPLS-DA	0.998	0.967	0.997	0.975
