# Supplementary material for: A Systematic Comparison of Differential Metabolites and Functional Pathways in the Gut Contents of Yaks and Plateau Yellow Cattle Across Altitudinal Gradients
Source: Vet Sci. 2026 Oct 9;13(10):1063. doi: 10.3390/vetsci13101063 (PMC13709441; doi:10.3390/vetsci13101063)
Supplement: Supplementary file 1 [file vetsci-13-01063-s001.zip › Supplementary Table S3.pdf]

Supplementary Table S3. Differentially abundant metabolites with concrete values

Metabolite/ID	log2FC	P value	FDR	Regulation	MSI level
M1000T309_3	1.59	8.25E-04	3.20E-03	Up	1–3
M1000T310	1.70	1.21E-02	2.91E-02	Up	1–3
M100T40	−1.59	5.01E-08	1.01E-06	Down	1–3
M100T94	−0.30	3.41E-04	1.55E-03	Down	1–3
M101T40	−2.15	1.87E-03	6.26E-03	Down	1–3
Thiiraneacetonitrile	−0.30	3.41E-04	1.55E-03	Down	1–3
Benzimidazole	−1.50	3.43E-04	1.56E-03	Down	1–3
4-Hydroxybut-2-ynal	0.79	1.71E-07	2.72E-06	Up	1–3
2-Methylbenzaldehyde	0.75	3.95E-08	8.33E-07	Up	1–3
(S)-2-Methylbutanal	0.54	3.81E-10	2.42E-08	Up	1–3
2-Aminoisobutyric acid	0.48	1.20E-07	2.72E-06	Up	1–3
