# Supplementary material for: A Systematic Comparison of Differential Metabolites and Functional Pathways in the Gut Contents of Yaks and Plateau Yellow Cattle Across Altitudinal Gradients
Source: Vet Sci. 2026 Oct 9;13(10):1063. doi: 10.3390/vetsci13101063 (PMC13709441; doi:10.3390/vetsci13101063)
Supplement: Supplementary file 1 [file vetsci-13-01063-s001.zip › Supplementary Table S4.pdf]

Supplementary Table S4. Enriched pathways with concrete values

Group	Pathway ID	Pathway	Total	P value	FDR
H vs L	ko04974	Protein digestion and absorption	47	1.65E-06	3.89E-04
H vs L	ko05230	Central carbon metabolism in cancer	37	1.06E-05	1.25E-03
H vs L	ko00403	Indole diterpene alkaloid biosynthesis	33	8.90E-05	5.68E-03
H vs L	ko00591	Linoleic acid metabolism	28	9.62E-05	5.68E-03
H vs L	ko00380	Tryptophan metabolism	83	9.73E-04	4.59E-02
H vs C	ko04974	Protein digestion and absorption	47	1.59E-08	4.00E-06
H vs C	ko05230	Central carbon metabolism in cancer	37	5.07E-06	5.78E-04
H vs C	ko04978	Mineral absorption	29	7.68E-05	5.84E-03
H vs C	ko00981	Insect hormone biosynthesis	25	7.22E-04	3.90E-02
H vs C	ko00410	beta-Alanine metabolism	32	9.68E-04	3.90E-02

Group	Pathway ID	Pathway	Total	P value	FDR
C vs L	ko04974	Protein digestion and absorption	47	1.00E-06	3.04E-04
C vs L	ko05230	Central carbon metabolism in cancer	37	2.00E-05	2.13E-03
C vs L	ko02010	ABC transporters	139	9.36E-04	3.60E-02
C vs L	ko00240	Pyrimidine metabolism	64	9.84E-04	3.60E-02
C vs L	ko00410	beta-Alanine metabolism	32	9.87E-04	3.60E-02
H vs L vs C	ko04974	Protein digestion and absorption	47	1.02E-03	9.44E-02
H vs L vs C	ko00403	Indole diterpene alkaloid biosynthesis	33	1.32E-03	9.44E-02
H vs L vs C	ko00073	Cutin, suberine and wax biosynthesis	27	1.73E-03	9.44E-02
H vs L vs C	ko00591	Linoleic acid metabolism	28	2.18E-03	9.44E-02
H vs L vs C	ko02010	ABC transporters	139	2.60E-03	9.44E-02
